# Traumatic Pelvic Ring Injury following Childbirth with Complete Pubic Symphysis Diastasis

**DOI:** 10.1155/2019/1785167

**Published:** 2019-11-15

**Authors:** Aaron Seidman, Kelley Brossy, Alfred Faulkner, Jeffrey Taylor

**Affiliations:** ^1^Department of Orthopedic Surgery, Beaumont Hospital, Farmington Hills, MI, USA; ^2^Lake Erie College of Osteopathic Medicine, Erie, PA, USA

## Abstract

**Case:**

Traumatic pelvic ring injury following childbirth is a rare but debilitating condition. We present a case of a 28-year-old female who sustained a traumatic pelvic ring injury following childbirth with a complete pubic symphysis separation of 5.6 cm treated successfully with nonoperative management.

**Conclusion:**

Operative and nonoperative treatments for traumatic pelvic ring injuries following childbirth have been described without universal adoption of a uniform treatment modality. We hope this case study adds to the collection of data to help guide medical decision-making in the future as surgeons encounter patients with similar orthopedic injuries.

## 1. Introduction

Traumatic pelvic ring injury with complete pubic symphysis diastasis following childbirth via vaginal delivery is a rare but debilitating condition. Widening of the cartilaginous joint during pregnancy prior to childbirth is physiologic and assists in widening the birth canal for successful delivery [1]. However, reports of nonphysiologic pubic diastasis exceeding that required for childbirth (typically greater than 1 cm) can leave mothers with debility and extreme pain. Incidence of complete separation of the pubic symphysis is reported to be within 1 in 300 to 1 : 30,000, with many instances likely undiagnosed [1, 2]. The orthopedic surgeon is presented with a difficult decision when managing these injuries as women are high-risk surgical candidates in the peripregnancy state, and prolonged debility can affect care for their newborn. We present the following case report of a 28-year-old female who sustained a traumatic pelvic ring injury with complete pubic diastasis of 5.6 cm that was successfully treated with nonoperative management, returning to full function one year out from injury. We present this case report in hopes to provide information to orthopedic surgeons presented with this challenging and debilitating diagnosis.

## 2. Statement of Informed Consent

Our patient was informed that data pertaining to her case and treatment would be submitted for publication and she agreed.

## 3. Case Report

Our patient is a 28-year-old G2PO female who presented to our institution in labor for the birth of her first child. At time of presentation, the position of the baby was cephalic. The patient denies antecedent pelvic pain or difficulty with ambulation prior to delivery. Active labor was initiated with Pitocin augmentation. She was provided an epidural spinal anesthesia. Following 3 hours of pushing, the patient delivered a baby boy of 6 lb 11.2 oz (3040 grams). The patient did sustain a grade 1 peroneal tear which was closed primarily with suture.

**Figure 1:**
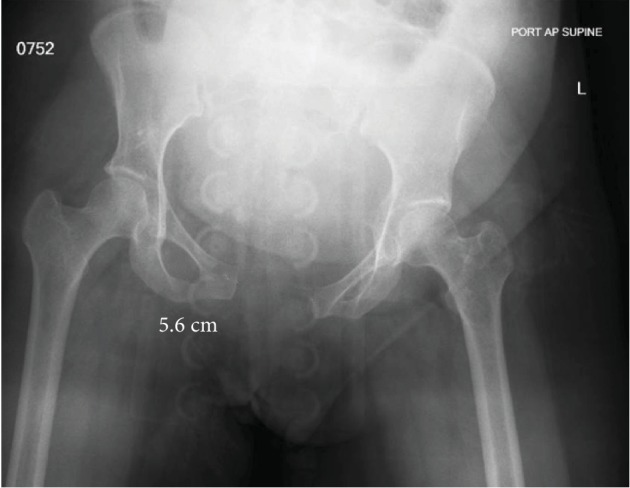
Diagnostic AP pelvis radiograph demonstrating a traumatic pubic ring separation of 5.6 cm.

**Figure 2:**
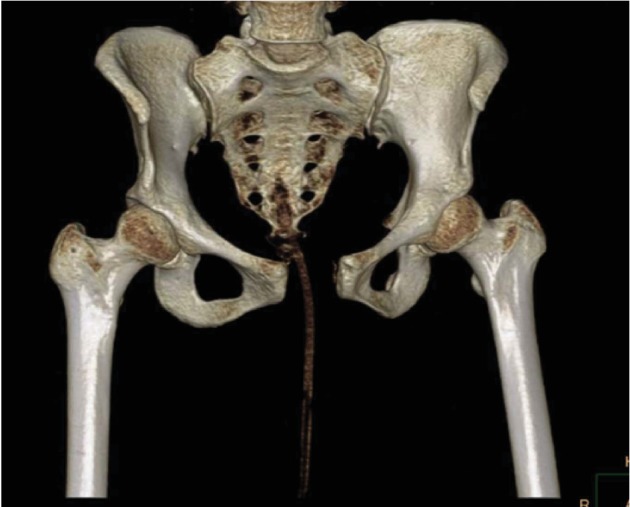
3D CT reconstruction of the pelvis redemonstrating separation of the pubic symphysis.

Two hours following delivery, the patient was evaluated by her obstetrics physician for persistent and worsening anterior pelvic pain and low back pain with inability to ambulate. She was then evaluated with an AP radiograph of the pelvis which revealed a complete pubic symphysis diastasis of 5.6 cm with widening of bilateral sacroiliac joints posteriorly (Figure 1) which is redemonstrated with 3D CT reconstruction (Figure 2). That evening, the orthopedic surgery team was asked to evaluate the patient. Following evaluation, a generic pelvic binder was placed on the patient and repeat imaging showed no significant improvement in her diastasis nor did the patient experience a reduction in her pain. The patient was left in a pelvic binder with constant skin assessments and was allowed to weight bear as tolerated. Considerations were given for both operative open reduction with anterior internal plate fixation and continued nonoperative management; however, the patient and family elected to continue nonoperative management with close observation.

**Figure 3:**
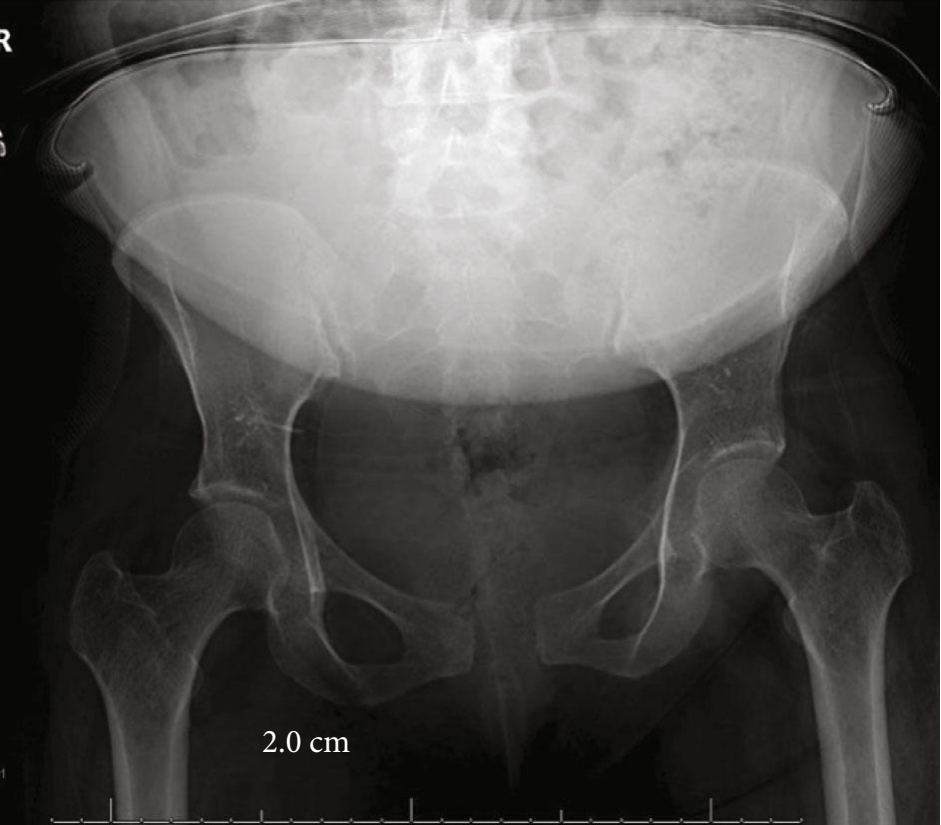
Follow-up AP pelvis radiograph at 6 weeks post injury demonstrating residual pubic symphysis widening of 2.0 cm.

The patient was transferred to the orthopedic surgery floor of our institution the following day where she worked with physical therapy twice daily for assistance with mobilization, beginning the following day post diagnosis. The patient had persistent pelvic pain and difficulty ambulating. On hospital day 8, as patient was only able to sit on the side of the bed and ambulate several feet with the assistance of a walker device, she was transferred to inpatient rehabilitation where she spent 15 days before being discharged home able to walk with minimal pain with assistance of the walker device. The patient was seen back in office for her first clinic follow-up 6 weeks following her delivery. At that time, the patient's AP pelvic X-ray revealed 2.0 cm of residual pubic diastasis (Figure 3), and the patient was using an occasional walker when on uneven ground but able to do stairs and perform ADLs. She continued to work with outpatient physical therapy for a total of 6 months before returning to full-time work. At her 1-year follow-up, she is back to full-time work, ambulates both inside and outside the home without assistance, and is able to do stairs, perform ADLs, and care for her baby with only mild intermittent low back pain managed by over-the-counter anti-inflammatories.

## 4. Literature Review

There is currently much debate over the appropriate treatment modality for postpartum pubic symphysis diastasis. Kharrazi et al. described four patients with an average pubic symphysis diastasis of 6.4 cm all treated nonoperatively with a pelvic binder. These patients had a reduction of their diastasis to 1.7 cm; however, all four had persistent sacroiliac joint pain. They suggest considering operative treatment at a diastasis of >4 cm. Dunivan described the use of an external fixator successfully on a woman with a 6.2 cm diastasis with the ability to weight bear on her second post-op day and discharged on postpartum day four, able to ambulate with a walker.

Cases of open reduction internal fixation (ORIF) of pubic symphysis diastasis have also been described in the literature. Najibi et al. describe ten patients treated operatively with internal fixation [3]. Three patients had an excellent outcome, four had a good result, and three had a fair or poor result. Rommens described the successful internal fixation of three patients with diastasis ranging from 15 mm to 45 mm who failed conservative management. Yoo et al. identified that primigravid females had a higher risk for diastasis.

Physical therapy for the management of pubic symphysis diastasis has been well documented. Stretch and often complete rupture of both the superior and inferior pubic ligaments result in separation of the symphysis. Strengthening of the surrounding soft tissue, including the rectus abdominis, thoracolumbar musculature and fascia, and quadriceps and hamstrings, helps stabilize and reduce stress through the pubic symphysis [4]. A stable symphysis without recurrent stress through the joint allows a slow return towards anatomic position with scarring of the torn ligaments back towards their attachments. A combination of closed chain exercises targeting these muscle groups along with altered sleep positioning (pillow between legs during slumber) are recommended to expedite the healing process [5].

## 5. Discussion

Widening of the pubic symphysis during childbirth is physiologic and is an advantageous adaptation in widening of the birth canal for delivery. However, excessive widening of the pubic symphysis can be pathologic and lead to debilitating pain. A separation of more than 1 cm postpartum is historically noted to be pathologic and symptomatic [1]. Furthermore, literature suggests consideration of operative treatment for separations greater than 4 cm [6]. Relaxin, a hormone secreted by the placenta during pregnancy, peaks during the first trimester and again peripartum. A modulator of arterial compliance and cardiac output during pregnancy, relaxin also serves to relax the pelvic ligaments and contributes to softening of the cartilage of the pubic symphysis for preparation of the birth canal for delivery [7, 8]. Identified risk factors for postpartum pubic symphysis diastasis include primigravid women, multiple gestations, and prolonged active labor [2]. When considering operative management of postpartum symphysis diastasis, it is important to consider and respect the physiologic changes of pregnancy that could complicate surgery. Pregnancy and peripartum bed rest are associated with an increased risk of deep venous thrombosis [2]. In addition, pelvic anatomy can be distorted following birth and elevated relaxin levels have also been shown to be associated with increased uterine bleeding, which complicate surgical treatment [2, 9]. Treatments described for pelvis diastasis include nonoperative treatment with application of pelvic binder coupled with physical therapy and immediate weight bearing, non-weight bearing with bedrest, closed reduction with application of binder, application of anterior external fixator with or without sacroiliac screw fixation, and anterior internal fixation with plate and screws. While our patient initially presented with a diastasis of 5.63 cm, we pursued nonoperative management with application of a pelvic binder and immediate physical therapy with unrestricted weight bearing. At 6-week follow-up, repeat imaging showed improvement of diastasis to 2.0 cm with significant improvement in symptoms. At 1-year follow-up, our patient is ambulating without assistance and is back to performing all activities of daily living and caring for her child.

Prognosis is good for the majority of patients who experience postpartum pubic symphysis diastasis, and in most cases, full recovery without persistent pain is expected [1]. Follow-up radiographs in most case studies reviewed show near complete closure of the pubic symphysis and complete resolution of symptoms within 3 months. Some patients did require further physical therapy for up to 6 months including our patient presented above. No significant long-term sequelae have been identified. No definitive recommendations exist regarding alteration of care for future pregnancies, and this would be a good area for future study. We hope this case study provides insight for future treating physicians.
